# Assessment of the proliferation status of glioblastoma cell and tumour tissue after nanoplatinum treatment

**DOI:** 10.1371/journal.pone.0178277

**Published:** 2017-05-31

**Authors:** Marta Kutwin, Ewa Sawosz, Slawomir Jaworski, Mateusz Wierzbicki, Barbara Strojny, Marta Grodzik, André Chwalibog

**Affiliations:** 1 Warsaw University of Life Science, Faculty of Animal Science, Department of Animal Nutrition and Biotechnology, Warsaw, Poland; 2 University of Copenhagen, Faculty of Health and Medical Sciences, Department of Veterinary and Animal Sciences, Frederiksberg, Denmark; Duke University School of Medicine, UNITED STATES

## Abstract

Glioblastoma is one of the most frequent primary brain tumours of the central nervous system, with a poor survival time. With inefficient chemotherapy, it is urgent to develop new strategies for tumour therapy. The present approach is based on the inhibition of cell proliferation using platinum nanoparticles (NP-Pt). The aim of the study was to evaluate and compare the antiproliferative properties of NP-Pt and cisplatin against U87 and U118 glioma cell lines and U87 tumour tissue. NP-Pt and cisplatin were incubated with U87 and U118 glioma cells or administered directly into glioma tumour tissue. Cell morphology, the level of DNA synthesis, the migration of cells, protein expression levels of proliferating cell nuclear antigen (PCNA) and the level of DNA oxidation in glioma tumours were investigated. The results showed that NP-Pt treatment of U87 and U118 glioma cells decreased the level of DNA synthesis and the migration of cancer cells but also downregulated the level of PCNA protein expression in tumour tissue. Furthermore, NP-Pt caused oxidative DNA damage in tumour tissue to a higher degree than cisplatin. Consequently, NP-Pt can be considered as an effective inhibitor of glioblastoma tumour cell proliferation. However, the mechanism of action and potential side effects need to be elucidated further.

## Background

Glioblastoma multiforme tumour (GBM) is the most frequent and malignant brain tumour (WHO grade IV) in adults, with a poor prognosis. The etiologic features of this central nervous tumour are still unknown. Therapeutic treatments based on radio- and chemotherapy do not significantly improve the survival rates of patients diagnosed with glioma [1]. Only the radiotherapy plus temozolomide improved the survival rates of glioblastoma patients. The major drawbacks of glioma treatments are the rapid infiltrating growth of tumour tissue, the ability to migrate and invasive tumour growth [2, 3]. Glioma cells are also able to degrade the extracellular matrix, stimulate cell invasion signalling pathways and thus invade healthy brain tissue [3]. Moreover, the proliferation of glioma cells is correlated with a high degree of tumour malignancy, which can be evaluated by measuring the protein expression of proliferating cell nuclear antigen (PCNA) [4]. Despite the novel strategy of treatments based on surgical resection and the combination of chemotherapy with radiotherapy, the main mechanisms of invasion, proliferation and migration in tumour cells are still not well elucidated. A better understanding of the proliferation and growth of glioma cells might offer a new therapeutic strategy involving the use of a new type of bioactive molecules; nanoparticles. To increase the efficiency of anticancer therapy, new approaches to the inhibition of cancer cell proliferation and malignancy using nanostructures are under investigation [5]. Nanoparticles are defined as small (<100 nm) particles with unique physicochemical properties. Recently, the application of nanoparticles has been considered as a new approach for the treatment and diagnoses of glioblastoma due to their catalytic activity, limited distribution of ions in the organism and possibilities for accumulation in glioma cells. Thus, the process of forming platinum salts with body fluids is very slow and restricted. Nanoparticles of noble metals, as NP-Pt, have a high surface-to-volume ratio, and are ideally suited as catalysts. Comparing to bare materials, NP-Pt require less energy activation than platinum metal. Moreover, NP-Pt catalyse chemical reaction including hydrogen evolution reaction and separating water into oxygen and hydrogen. The antioxidative properties of NP-Pt, where NP-Pt inhibited hydrogen peroxide and induced oxidative cellular damage in HepG2^6^ have been demonstrated [6]. Moreover, NP-Pt are able to cross the cell membrane and accumulate in glioma cells [7]. NP-Pt (99,999%) with no coating and/or stabilization additives, like *e*.*g*. PVP, have larger active surface area making nanoparticles more biologically active at lower doses [6] and consequently may affect their antiproliferative activity.

Platinum nanoparticles (NP-Pt) showed dose-dependent toxicity against different types of human cancer cells including breast MCF-7, liver HepG-2 [6], colorectal HT29 [8], lymphoma U937 [9] and glioma U87 [6], U251 [10]. Moreover, NP-Pt combined with hadron cancer therapy created DNA strand breaks and activated apoptosis in HT29 cancer cells [8]. The direct interaction of NP-Pt with the double helix of DNA, the formation of DNA strand breaks and consequent activation of the apoptosis pathway in cancer cells were also observed in U87 glioma cells [6], Caco-2 [11], HT29 [12] colon carcinoma cells and C6 rat glioma [13]. Toxicity investigations with the *in vivo* mice model, demonstrated that NP-Pt, but with size less that 1nm, induced the kidney injury after i.v. administration [14], and also can induce the mitochondria degradation of brain tissue samples, activation of apoptosis and reduced rate of the brain cell proliferation [15]. However, these side effects had a minor influence on general health parameters and were less toxic comparing to the side effect of cisplatin, including drug resistance, haemolysis, nephrotoxicity, ototoxicity, hepatotoxicity and blood marrow damage [16].

Despite increased numbers of scientific reports about a biointeraction between NP-Pt and various lines of cancer cells, the effect of NP-Pt on the proliferation and migration of glioblastoma cells is still not well elucidated. Moreover, until now, there has been insufficient data regarding the inhibition of proliferating cell nuclear antigen (PCNA) expression by NP-Pt at U118 and U87 glioblastoma cells and tumour tissue. The direct comparison of antiproliferative features of NP-Pt vs. cisplatin against U87 and U118 or any other glioblastoma cells line have not been documented so far. Thus, this study was designed to compare the anti-proliferative efficiency of two different sources of Pt against two genetically and morphological different cell lines of glioblastoma—U87 and U118.

We hypothesized that because of unique physicochemical features of NP-Pt, nanoplatinum may exert, different from cisplatin treatment, antiproliferative effects on U118 and U87 glioblastoma cells and tumour. The novel strategies of glioblastoma treatments are based on the previous achievements, including chemotherapy, but also radiotherapy and very precise resection of glioblastoma tumour. According to *clinicaltrails*.*go*v, there are only three clinical trials for glioblastoma treatment using nanotechnology, but only one of them have same results from Phase I. Consequently, we suppose the presented approach provides some relevant and novel results.

The objective of the present study was to determine the antiproliferative efficiency of NP-Pt, in comparison with cisplatin, on glioblastoma cell lines and tumour tissue.

## Materials and methods

### Ethics statement

The experimental procedures were performed in accordance with Polish legal regulations concerning experiments on animals (Dz. U. 05.33.289). The experimental protocols were accepted by the III Local Ethics Commission for Experimentation on Animals at Warsaw University of Life Sciences, Poland.

### Preparation and characterisation of nanocolloids

The colloid of nanoparticles of platinum was purchased from Nano-koloid (Warsaw, Poland). This material is produced by a patented electric non-explosive method (Polish patent 380649) from high-purity metal (99.9999%) and high-purity demineralised Milli-Q water. After 30 minutes of sonication, the solution was diluted to different concentrations with 1× Dulbecco’s modified Eagle’s culture medium (Sigma–Aldrich, St. Louis, MO, USA), immediately prior to exposure to GBM cells or tumour tissue.

The shape and size of the NP-Pt were inspected using a JEM-1220 (JEOL, Tokyo, Japan) transmission electron microscope (TEM) at 80 KeV, with a Morada 11-megapixel camera (Olympus Soft Imaging Solutions, Münster, Germany). Samples for the TEM were prepared by placing droplets of hydrocolloids on to Formvar-coated copper grids (Agar Scientific, Stansted, UK). Immediately after drying of the droplets in dry air, the grids were inserted into the TEM.

The zeta potential in water was measured using a Zetasizer Nano ZS model ZEN3500 (Malvern Instruments, Malvern, UK). The test was performed in triplicate.

Cisplatin (cis-diamineplatinum (II) dichloride) was obtain from Sigma (479306; Sigma, St. Louis, MO, USA) and diluted to 20 μg/ml in ultrapure Milli-Q water, and it was diluted to different concentrations with 1× Dulbecco’s modified Eagle’s culture medium (Sigma–Aldrich, St. Louis, MO, USA), immediately before use. The concentration of NP-Pt was equal to the atomic mass of Pt atoms in cisplatin. The concentration of NP-Pt was based on published data about anticancer properties of cisplatin [16].

### Cell cultures and treatments

Human glioblastoma U118 and glioblastoma likely U87 cell [17, 18] lines were obtained from the American Type Culture Collection (Manassas, VA, USA) and maintained in Dulbecco’s modified Eagle’s culture medium supplemented with 10% foetal bovine serum (Sigma-Aldrich), 1% penicillin, and streptomycin (Sigma-Aldrich) at 37°C in a humidified atmosphere of 5% CO2/95% air in a NuAire DH AutoFlow CO2 Air-Jacketed Incubator (Plymouth, MN, USA).

### Cell morphology

U87 and U118 glioma cell lines were placed on 6-well plates (1x 10^5^ cells per well) and treated with NP-Pt and cisplatin after overnight incubation. After 24h of incubation, the morphology of cells was recorded under an optical microscope (DM750; Leica Microsystems GmbH, Wetzlar, Germany) using LAS EZ version 2.0 software. The experiment was performed in three repetitions (each experimental group in triplicate).

### Proliferation assay by BrdU incorporation

Cell proliferation was evaluated using a bromodeoxyuridine (BrdU) incorporation assay (BrdU colorimetric) (Roche Applied Science, Indianapolis, IN, USA). U87 and U118 cell lines (1×10^4^) were placed in 96-well plates and cultured overnight. After incubation, medium was removed and hydrocolloids of NP-Pt and cisplatin were introduced to the cells for the next 24 h. Then, the culture media was removed, the cells were fixed, and the DNA was denatured in one step by adding FixDenat. In the next step, the cells were incubated with anti-BrdU-POD antibody for 90 minutes at room temperature. After the removal of the antibody conjugate, the cells were washed and the substrate solution was added. The reaction product was quantified by measuring absorbance using a scanning multi-well spectrophotometer (Infinite M200, Tecan, Durham, NC, USA) at 370 nm with a reference wavelength of 492 nm and finally expressed as the difference between BrdU-positive and -negative samples, expressed as Optical Density (O.D.). All experiments were performed in three repetitions (each experimental group in triplicate).

### Cell migration assay

The cell migration assays were performed using 8-μm pore inserts (Starstedt, Nümbrecht, Germany) pre-coated with 0.5 μg/ml of the chemo-attractant fibronectin (Sigma). U87 cells (5 × 10^4^) were placed into transwell inserts in triplicate and incubated overnight. Next, the cells were treated with NP-Pt hydrocolloid and cisplatin solution and allowed to migrate for 24h.

Migrated cells were fixed with 4% paraformaldehyde (Sigma–Aldrich) in PBS and visualised by staining with 0.1 μg/ml DAPI (Life Technologies, Gaithersburg, MD, USA). Migration was determined by imaging DAPI nuclear stain and viewed using a CKX 41 epifluorescent inverted microscope (Olympus, Tokyo, Japan) with a fluorescent filter, and the image was captured with a ProgRes c12 camera (Jenopik, Germany). The migration experiment was performed in three repetitions (each experimental group in triplicate). The number of cells that had passed through the membranes were counted as a measure of their migration potential.

### Culture of GBM on chorioallantoic membrane

Fertilised eggs (Gallus gallus) (n = 60) were supplied by a commercial hatchery. After 6 days of egg incubation, the silicone ring with the deposited 3–4 × 10^6^ U87 cells suspended in 30 μL of culture medium was placed on the chorioallantoic membrane according to the procedure described by. Grodzik et al. [19]. The eggs were incubated for 7 days; then 36 eggs with visible tumour development were chosen. Eggs were divided into three groups of 12: the control group, 8.45μM/mL NP-Pt group (injected with 200 μL of solution) and 13μM/mL cisplatin group (injected with 200 μL). The solutions were slowly injected with insulin syringe (Ø = 29 mm) under sterile conditions in a laminar chamber. The dose of cisplatin for direct intratumour injection was calculated based on Cemazar et al. [20]. The concentration of NP-Pt was equal to the atomic mass of Pt atoms in cisplatin. The solutions were added directly into the tumours. After 2 days, the tumours were resected for further analysis.

### Examination of proliferation of tumour tissue by PCNA protein expression

The proliferating cells were identified *via* immunofluorescence and immunohistochemistry, using antibody directed against proliferating cell nuclear antigen (PCNA). Glioma tumour tissue samples from each group (n = 3 x 4) were collected and fixed for immunofluorescent and immunohistochemistry analyses, according to standard procedures described by Prasek et al. [15] and Urbanska et al.[21]. For fluorescence analysis, tissue samples were washed with PBS and permeabilised with 0.5% Tween 20 (Sigma-Aldrich) PBS solution for 10 min. The sections were blocked with PBS containing 2% of goat serum and 1% bovine serum albumin (Sigma-Aldrich) for 30 min. Sections were incubated with primary rabbit anti-PCNA antibody (No. 15580, Abcam, GB) diluted in 2% goat serum for 16 h at 4°C (according to the manufacturer's instructions). After PCNA localisation in gliomas tumour tissue washing sections were incubated with secondary antibody: goat anti-rabbit Atto 488 conjugate (IgG (H+L),F(ab’)2 Fragment Atto488; Cell Signaling Technology, Danvers, USA) for 1 hrs, diluted according to producer instructions. Nuclei were stained by incubation with 4′,6-diamidino-2-phenylindole (DAPI) solution for 15 min (Sigma-Aldrich). After washing, the cover slips were mounted on slides with Fluoromount mounting medium (Sigma-Aldrich) and observed on IX 81 FV-1000 confocal microscope (Olympus Corporation, Tokyo, Japan). Image analysis in confocal mode, Nomarski interference contrast and cell counting were performed using FVIO-ASW ver. 1.7c software (Olympus). Three-dimensional images were assembled from 30 optical sections. The sections for immunohistochemistry investigation were incubated for 1 hour with the rabbit polyclonal anti- PCNA at room temperature, and visualised with DAKO EnVision+System-HRP (K 4010, DAKO, Glostrup, Denmark). The proliferation levels were expressed as the number of PCNA-positive cells in the tumour sections (counted area– 3500 μm^2^).

### Analysis of 8-hydroxy-2'-deoxyguanosine (8-OH-dG) concentration in tumour tissue DNA

8-HO-dG is a modified base of nucleoside and it is mainly detected as a product of oxidative DNA damage. Samples of tumour tissue from each group (n = 3 x 4) were collected and frozen in -80°C. The samples were refrozen and 5 mL of homogenisation buffer (0.1 M phosphate buffer, pH 7.4, containing 1 mM EDTA) per gram of tissue was added. Next, the homogenisation procedures were conducted according to the commercial available protocol, using TissueLyser homogeniser (86500, Qiagen, Hilden, Germany). The samples were centrifuged at 1,000 x g for 10 minutes and the supernatants were purified using a commercially available DNA extraction kit (A1120, Promega, Madison, WI, USA).

8-OH-dG was measured in tumour tissue DNA samples, using a commercial 8-OH-dG ELISA kit (ab201734, Abcam, Cambridge, UK) following the manufacturer’s protocol. Samples were diluted at a ratio of 1:10 of a sample to the final concentration of DNA 19.6 ng/ml, the concentration of 8-HO-dG was measured at 450 nm.

### Statistical analysis

Data were analysed using one-way analysis of variance with Statgraphics^®^ Plus 4.1 (StatPoint Technologies, Warrenton, VA, USA). The differences between groups were tested using Tukey’s multiple-range tests. All mean values are presented with the standard deviation.

## Results

### NP-Pt characterisation

**Fig 1:**
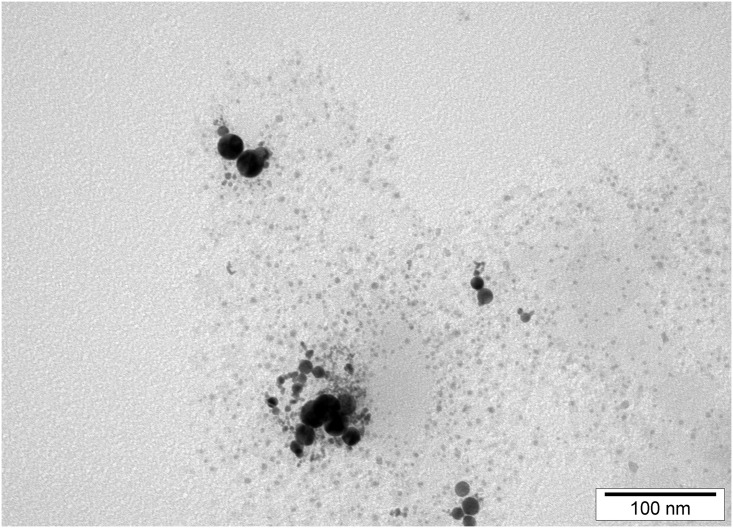
Transmission electron microscopic images of platinum nanoparticles. Bar scale 100nm.

Fig 1 shows TEM imagines of NP-Pt. NP-Pt had regular round shape and diameter of the particles ranged from 2 nm (>60%) to 25 nm (<5%) (Fig 1). The mean zeta potential of NP-Pt was -27.86 mV.

### Cell morphology

**Fig 2:**
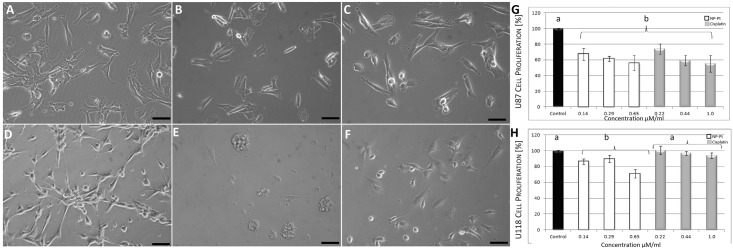
Cell morphology and BrdU incorporation assay. Optical microscopy images of platinum nanoparticles (NP-Pt) or cisplatin -treated and untreated U87 and U118 glioma cells. (A) U87 control; (B) NP-Pt—treated U87; (C) cisplatin-treated U87; (D) U118 control; (E) NP-Pt-treated U118; (F) cisplatin-treated U118. BrdU incorporation assay–(G)–U87 cells; (H)–U188 cells. ^a, b^ Values with different letters are significantly different, P < 0.05. Bar scale 100μm.

Evaluation of U118 and U87 glioma cells by light microscopy demonstrated that U87 and U118 gliomas cells were characterised by a different cells morphology (Fig 2). U118 cells had smaller and less branched protrusions than U87 cells. U118 were smaller and grew much slower than U87 cells. The administration of NP-Pt as well as cisplatin had harmful effects on gliomas cells morphology. In every cell line the NP-Pt treatment caused the cells membrane deformation and decreased the cells density. Cell morphology was similar in the NP-Pt and cisplatin groups, but the treated cells lost their characteristic morphology (branched protrusions, shape, size) in comparison to the control group.

### Cell proliferation

BrdU is a synthetic analogue of thymidine which during the cell cycle is implemented into DNA. The increased level of BrdU was equal to the increased cell proliferation. The U118 and U87 cells proliferation status detected by BrdU incorporation showed that increasing concentration of NP-Pt decreased the cell proliferation comparing to the control group (Table A in S1 File). According to the BrdU assay, NP-Pt had significant higher anti-proliferative activity comparing to cisplatin, especially against U118 cell line (Table B in S1 File). The bars form Fig 2G, indicate a significantly lower average of proliferating U118 cells. The comparison of the proliferation between the cell lines showed that U87 cells were more sensitive that U118 cells (Fig 2).

### Cell migration assay

**Fig 3:**
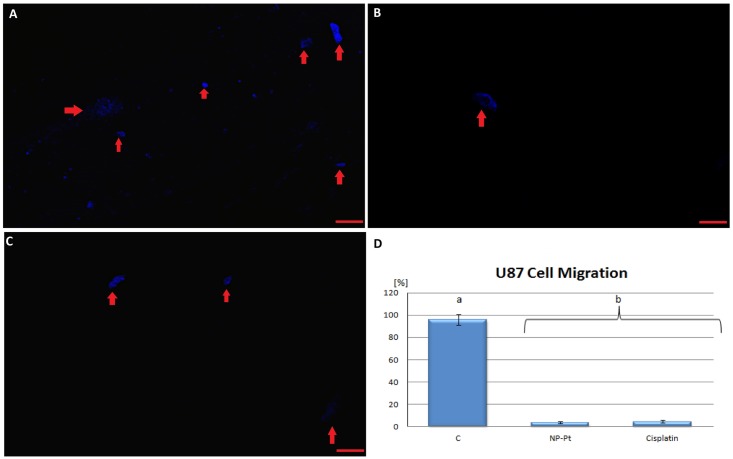
U87 cell migration. Confocal fluorescent microscopy images of platinum nanoparticles (NP-Pt) or cisplatin -treated and untreated U87 glioma cells. (A) U87 control; (B) NP-Pt—treated U87; (C) cisplatin-treated U87; (D) effect of NP-Pt on the migration of U87 glioma cells ^a, b^ Values with different letters are significantly different, P < 0.05. Bar scale 40μm. Note: red arrows—U87 glioma cells shown as an overlaid image of 4′,6-diamidino-2-phenylindole-stained nuclei (blue). Abbreviations: C—control group.

Comparison to U87 glioma cells from the control group showed that U87 glioma cells lost their ability for migration after NP-Pt administration (Fig 3). These results indicated that NP-Pt administration to U87 cells affects the migration of gliomas cells with comparable efficiency as cisplatin solution (Table D and Table E in S1 File).

### Tumour tissue proliferation assay

**Fig 4:**
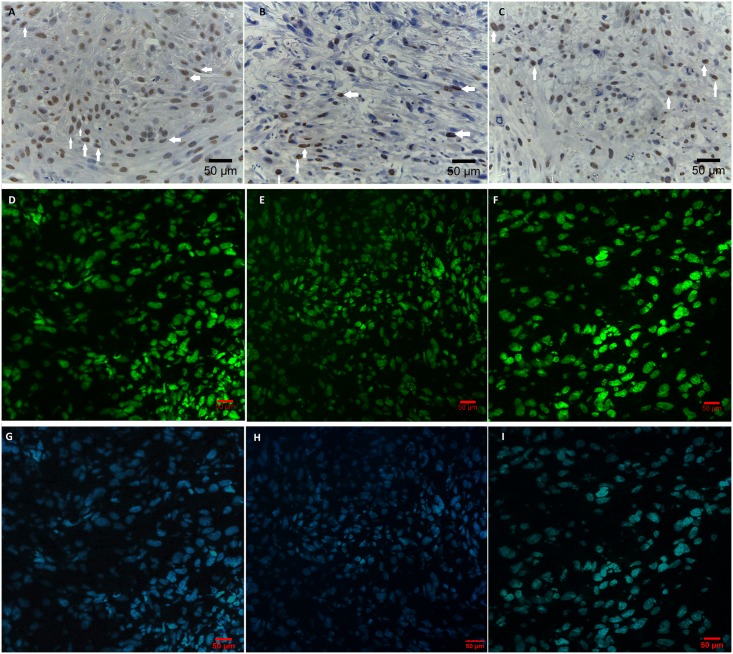
Protein expression level of PCNA at U87 glioma tumour tissue. Visualization of PCNA *via* immunohistochemistry (A, B, C) and immunofluorescence (D, E, F, G, H, I) in glioblastoma tumours. (A), (D), (G)- U87 control; (B), (E), (H)- NP-Pt—treated U87; (C), (F), (I)- cisplatin-treated U87. Scale bars: 50 μm. Note: white arrows point to PCNA positive nuclei.

The evaluation of proliferation status at tumour tissue showed that intratumoural injection of NP-Pt hydrocolloid deceased significantly the proliferation of tumour cells. The number of PCNA-positive cells decreased after NP-Pt administration comparing to the control (non-treated) group (Table 1). A comparison of cisplatin treatment and NP-Pt showed that the reduction of proliferation was lower in the cisplatin group (Table C in S1 File). These results indicate that the proliferation and density of tumour tissue were significantly reduced after NP-Pt treatment (Fig 4), (Fig A in S1 File).

**Table 1:** Numbers of PCNA positive nuclei in glioblastoma multiforme tumour tissue (area counted 3500 μm^2^) in the control group and in groups treated with platinum nanoparticles and cisplatin.

	Control	Platinum nanoparticles	Cisplatin	SE-pooled
Number of cells	199^a^	113^b^	153^b^	12.35

^a,b^ Within rows: means with different superscripts differ significantly (P<0.001)

### Analysis of 8-hydroxy-2'-deoxyguanosine (8-OH-dG) concentration in tumour tissue DNA

The evaluation of 8-OH-dG level in tumour tissue showed that intratumoural injection of NP-Pt hydrocolloid significantly increased concentration of 8-OH-dG in DNA (Fig B in S1 File, Table F in S1 File) comparing to the control (non-treated) and cisplatin group (Table 2). The result indicate that NP-Pt efficiently cause oxidative DNA damage in tumour tissue.

**Table 2:** Formation of 8-HO-dG in nuclear DNA of human gliblastoma multiforme tumour tissue cells treated with platinum nanoparticles and cisplatin.

	Control	Platinum nanoparticles	Cisplatin	SE-pooled
8-HO-dG concentration [pg/mL]	180.9^a^	383.2^b^	204.1^a^	14.78

^a,b^ Within rows: means with different superscripts differ significantly (P<0.0001)

## Discussion

The results of the present studies indicated that NP-Pt can be an efficient inhibitor of glioma cells proliferation. NP-Pt have fewer side effects on general health than do platinum salts such as cisplatin and localise primarily at target/specific cells or tissues [6, 21 – 22]. Moreover, the size <20 nm of NP-Pt determines their bioavailability for cells and tissue and improves the NP-Pt uptake by cancer cells [6–7, 9, 12, 22]. The inhibition of proliferation processes is crucial to patient treatment in the dynamic fields of oncology, especially neurooncology, where the downregulation proliferation of cancer cells is crucial for the success of treatment [23]. The obtained results showed that NP-Pt can affect proliferation of glioblastoma cells and tissue. The incorporation of BrdU into the DNA helix showed that NP-Pt treatment decreased the proliferation of U87 and U118 cells and also caused morphological deformations (dwindled cytoplasmic protrusions). Furthermore, this dose-dependent toxicity showed that NP-Pt treatment decreased cell migration, which affects the malignancy of U87 glioma tumours. In addition, evaluation of the migration potential of glioma cells provided information about the influence of NP-Pt on metastasis at tumour tissue, indicating that NP-Pt treatment, as well as cisplatin can reduce the metastasis of glioblastoma tumour cells by reducing the number of circulating tumour cells in the bloodstream. Our previous report demonstrated that NP-Pt had anticancer properties and increased the apoptosis of glioma cells and tumour tissue[6]. While in the present experiments, we demonstrated that NP-Pt and cisplatin decreased cell migration, indicating that both Pt treatments can reduce the metastasis of glioblastoma tumour cells by reducing the number of circulating cells in the bloodstream. NP-Pt were also able to arrest the cell cycle and caused apoptosis of U251 glioma cells [10]. However, the U251 and U87 glioblastoma cell lines are different regarding proliferation, invasion and migration ability. Moreover, comparing protein expression between U251 and U87 cell lines, the U251 has 263 proteins that have a lower level of expression comparing to the U87 line [24]. Therefore, these proteins are involved in a drug resistance, because the drugs that target the purine metabolism are more toxic to U251 than U87 cell line. Additionally, the U251, U87 and U118 have also different genetic mutations (p53, p21, CDKN2A, PTEN) which affect anticancer properties of platinum-based drugs.

The invasion of glioma cell into brain tissue structure is caused by interactions with extracellular matrix and modification of cancer cell morphology including prolongation of protrusions, which become an anchor and allowing interactions between glioma cells and cytoskeleton [24]. The results of *in vitro* experiments showed that NP-Pt treatment was as efficient as cisplatin in terms of changes in cell morphology, proliferation, and migration.

Platinum-based anticancer drugs such as cisplatin, carboplatin or oxaliplatin are commonly used to treat several types of cancer like: head and neck, lung, colorectal cancer and other solid tumours [25]. The main mechanism of the anticancer activity of platinum salts is based on the formulation of cisplatin-DNA adducts as a result of mismatch repair and cellular levels of damage-recognition proteins [26]. However, the major drawbacks of platinum-based cancer chemotherapy are neurotoxicity, nephrotoxicity, ototoxicity and limited efficiency caused by developed chemoresistance of cancer cells [27]. Recent studies showed that the intratumoural injection of NP-Pt decreased PCNA protein expression in glioma tumours. PCNA is a 36 kDa molecular weight non–histamine nuclear protein, which expression occurs only in proliferating cells. The increased level of PCNA expression is mainly observed during G1 and S phase of the cell cycle and contributes to DNA replication [4]. The evaluation of PCNA expression is significant in terms of the determination of malignancy, metastasis and tumour grade [28]. Moreover, PCNA is also the basic component of recombination-associated DNA synthesis during the process of double-strand break repair in chemoresistant cancer or after treatment with platinum-based cancer drugs [29–30]. Previous studies of PCNA protein expression levels in astrocytoma and oligoastrocytoma indicated that increased expression was correlated with increasing tumour grade [29] and decreasing patient survival rates [31–33]. In the present study, we found that the protein expression of PCNA was significantly lower in the treated tumour samples than in the control. Our results are similar to previous results about the anticancer properties and antiproliferative properties of nanoplatinum, where NP-Pt of 5–8 nm in size mediated cell growth arrest, downregulated PCNA protein expression and activated apoptosis in U251 glioma cells [10]. The observation of *in ovo* glioma model also showed that single-dose injection of NP-Pt stayed locally, and exudation of NP-Pt from tissue samples or general toxicity to chicken embryos was not detected. The *in ovo* model of glioblastoma tumour meets criteria for the xenograft model of glioblastoma including sufficiently similar protein expression panel comparing to the nude rat models [34], easy access to the tumour tissue, time controlled growth, and a low cost. Moreover, the *in vivo* models for brain tumour growth should be developed from glial cells, and their growth should be also predictable and reproducible. Strojnik et al. [34] described the *in ovo* and *in vivo* U87 glioblastoma tumour, and in both models, authors observed the typical cytological structure of glioma tumour including the presence of astrocytes, small anaplastic cells, spindle and giant cells. On the other hand, the major issue with *in ovo* models, is limited observation of infiltrative growth of glioblastoma tumour into brain structure. Currently, authors conduct investigations using *in vivo* rat model to confirm *in ovo* results, but the data still need to be elucidated in follow-up studies.

*In vivo* anticancer activity of NP-Pt was also observed in a glioblastoma rat model, where nanosized platinum had a negative impact on vascularisation and decreased tumour tissue growth [13]. The anticancer properties of NP-Pt induce DNA damage, caused by the interaction between the OH groups on the NP-Pt surface with phosphate groups on the DNA helix [12]. In the present study the evaluation of 8-HO-dG concentration after NP-Pt treatment demonstrated that NP-Pt have a strong affinity to DNA, thus, NP-Pt can be considered as a new type of antineoplastic agents. Furthermore, anticancer properties of NP-Pt involve cell-cycle inhibition by Pt^2+^ ions released from NP-Pt after interactions between NP-Pt and hydrogen peroxide in endosomes [10,12]. The main mechanism of proliferation inhibition by NP-Pt may be caused by the bidirectional interaction of Pt^+^ ions with the cytoskeleton of cancer cells or the formation of DNA strand breaks [6–7]. The results, also pointed on the antiproliferative properties of NP-Pt, caused by degradation of NP-Pt surface and release of Pt+ ions, which may generate the oxidative stress, affect the DNA damage and consequently decrease the proliferation of tumour glioma cells. The results of the present study on U87 and U118 glioma cell lines and tumour tissue are in agreement with previous studies with other cancers, demonstrating that NP-Pt treatment has antiproliferative properties against HT29 human colon carcinoma [8], SiHa cervical cancer [35] and U251 glioblastoma [10]. The possible mechanism of action of NP-Pt against U87 and U118 glioblastoma cells is mainly related to DNA damage by NP-Pt attachment into DNA and formation of DNA strand breaks, which was confirmed by the production of 8-OH-dG in glioma tumour tissue. Moreover, this mechanism is directly correlated with the decrease of PCNA activity and consequently the decrease of cell proliferation, caused probably by arrest of DNA synthesis at S phase of cell cycle. Comparing the antiproliferating efficiency of cisplatin against U87 the *in vitro* investigation showed that NP-Pt had a better antiproliferative efficiency than cisplatin. The *in ovo* measurements also revealed that NP-Pt had a better antiproliferating efficiency against U87 tumour tissue than cisplatin, at the same concentration of Pt atoms. Thus, the results indicate that NP-Pt may be used in glioblastoma cancer therapy as inhibitors of tumour cell proliferation, but the mechanism of action and potential side effects, as well as glioma cells line characterisation, need to be elucidated in the follow-up research.

## Conclusions

The *in vitro* investigation demonstrated that NP-Pt had antiproliferative effects on U87 and U118 glioma cells. The U87 glioma cells treated with NP-Pt showed morphological deformations, decreased proliferation, and were more sensitive to NP-Pt treatment than U118 glioma cells. The *in ovo* studies showed that NP-Pt treatment decreased PCNA protein expression level. The measurements also revealed that NP-Pt caused oxidative DNA damage in tumour tissue to a higher degree than cisplatin. The results indicate that NP-Pt may be used in glioblastoma cancer therapy. However, potential side effects must be elucidated in *in vivo* future research.

## Supporting information

S1 File (DOC)
