# Supplementary material for: Assessment of the proliferation status of glioblastoma cell and tumour tissue after nanoplatinum treatment
Source: PLoS One. 2017 May 31;12(5):e0178277. doi: 10.1371/journal.pone.0178277 (PMC5451066; doi:10.1371/journal.pone.0178277)
Supplement: S1 File — (DOC) [file pone.0178277.s001.doc]

**S1 Table A. Proliferation assay by BrdU incorporation.** Percentage of incorporated BrdU into DNA helix.

| **Proliferating cells**  **Group** | **U87 [%]** | **U118 [%]** |
| --- | --- | --- |
| Control | 99 | 100 |
| Control | 100 | 101 |
| Control | 98 | 99 |
| NP-Pt 0.14 | 60 | 86 |
| NP-Pt 0.14 | 75 | 88 |
| NP-Pt 0.14 | 67 | 85 |
| NP-Pt 0.29 | 59 | 89 |
| NP-Pt 0.29 | 60 | 90 |
| NP-Pt 0.29 | 65 | 88 |
| NP-Pt 0.65 | 61 | 71 |
| NP-Pt 0.65 | 45 | 69 |
| NP-Pt 0.65 | 62 | 70 |
| CisPt 0.22 | 80 | 100 |
| CisPt 0.22 | 74 | 99 |
| CisPt 0.22 | 67 | 99 |
| CisPt 0.44 | 66 | 96 |
| CisPt 0.44 | 59 | 96 |
| CisPt 0.44 | 53 | 95 |
| CisPt 1.0 | 66 | 94 |
| CisPt 1.0 | 45 | 93 |
| CisPt 1.0 | 55 | 95 |

Abbreviations: NP-Pt – platinum nanoparticles, CisPt – cisplatin.

**S1 Table B. Percentage average of BrdU incorporation.**

| **BrdU assay** |  | **Control** | **NP-Pt 0.14** | **NP-Pt 0.29** | **NP-Pt 0.65** | **CisPt 0.22** | **CisPt 0.44** | **CisPt 1.0** |
| --- | --- | --- | --- | --- | --- | --- | --- | --- |
|  | U87 [%] | 100 | 67.3 | 61.3 | 56 | 73.6 | 59.3 | 55.3 |
|  | St.Dev. [%] | 1.0 | 7.5 | 3.2 | 9.5 | 6.5 | 6.5 | 10.5 |
|  | U118 [%] | 99 | 86.07 | 89.52 | 70.8 | 100.3 | 96.51 | 93.9 |
|  | St.Dev.[%] | 1.5 | 3.6 | 4.4 | 5.3 | 5.3 | 2.5 | 3.5 |

Abbreviations: NP-Pt – platinum nanoparticles, CisPt – cisplatin, St. Dev. - standard deviation

**S1 Table C. Number of PCNA positive cells.**

| **Group** | **Number** | **Average number of cells** | **St.Dev.** |
| --- | --- | --- | --- |
| Control | 193 | 199 | 29.6 |
| Control | 259 |
| Control | 210 |
| Control | 172 |
| Control | 177 |
| Control | 182 |
| Control | 202 |
| NP-Pt | 88 | 113 | 25.5 |
| NP-Pt | 133 |
| NP-Pt | 140 |
| NP-Pt | 142 |
| NP-Pt | 87 |
| NP-Pt | 110 |
| NP-Pt | 88 |
| CisPt | 162 | 153 | 34.9 |
| CisPt | 129 |
| CisPt | 174 |
| CisPt | 115 |
| CisPt | 218 |
| CisPt | 132 |
| CisPt | 144 |

Abbreviations: NP-Pt – platinum nanoparticles, CisPt – cisplatin, St. Dev. - standard deviation

**S1 Table D. Migration assay. Number of migrating cells.**

| **Migration Assay** | **Number of cells** | | | | | | | | | |
| --- | --- | --- | --- | --- | --- | --- | --- | --- | --- | --- |
| Control | 16 | 16 | 6 | 9 | 1 | 6 | 9 | 21 | 17 | 5 |
| Control |  | 10 | 7 | 16 | 3 | 10 | 13 | 14 | 14 | 11 |
| Control | 12 |  | 9 |  |  | 14 | 11 |  | 5 | 7 |
| Control | 2 | 3 | 3 | 5 | 16 | 31 | 10 | 18 | 5 | 12 |
| NP-Pt | 1 | 0 | 1 | 2 | 2 | 1 | 0 | 0 | 12 | 10 |
| NP-Pt | 5 | 0 | 1 | 4 | 3 | 14 | 11 | 1 | 1 | 0 |
| NP-Pt | 5 | 0 | 1 | 0 | 1 | 2 | 1 | 5 | 9 | 4 |
| NP-Pt | 0 | 2 | 2 | 20 | 3 | 0 | 5 | 0 | 0 | 16 |
| CisPt | 7 | 2 | 1 | 4 | 2 | 0 | 2 | 13 | 0 | 3 |
| CisPt | 2 | 1 | 0 | 12 | 3 | 8 | 8 | 15 | 15 | 0 |
| CisPt | 0 | 1 | 6 | 3 | 1 | 12 | 11 | 2 | 0 | 1 |
| CisPt | 12 | 8 | 4 | 3 | 3 | 5 | 3 | 0 | 8 | 3 |

Abbreviations: NP-Pt – platinum nanoparticles, CisPt – cisplatin.

**S1 Table E. Statistic summary of migration assay.** Percentage of cells migration.

| Group | [%] Average | St.Dev |
| --- | --- | --- |
| Control | 95.6 | 4.83 |
| NP-Pt | 3.6 | 1.04 |
| CisPt | 4.6 | 1.44 |

Abbreviations: NP-Pt – platinum nanoparticles, CisPt – cisplatin, St. Dev. - standard deviation.

**S1 Fig A. Protein expression level of PCNA at U87 glioma tumour tissue**. Visualization of PCNA *via* immunofluorescence (D, E, F, G, H, I) in glioblastoma tumors.  *(D), (G)-* U87 control; (E), (H)- NP-Pt – treated U87; (F), (I)- cisplatin-treated U87*.* Scale bars: 50 μm.


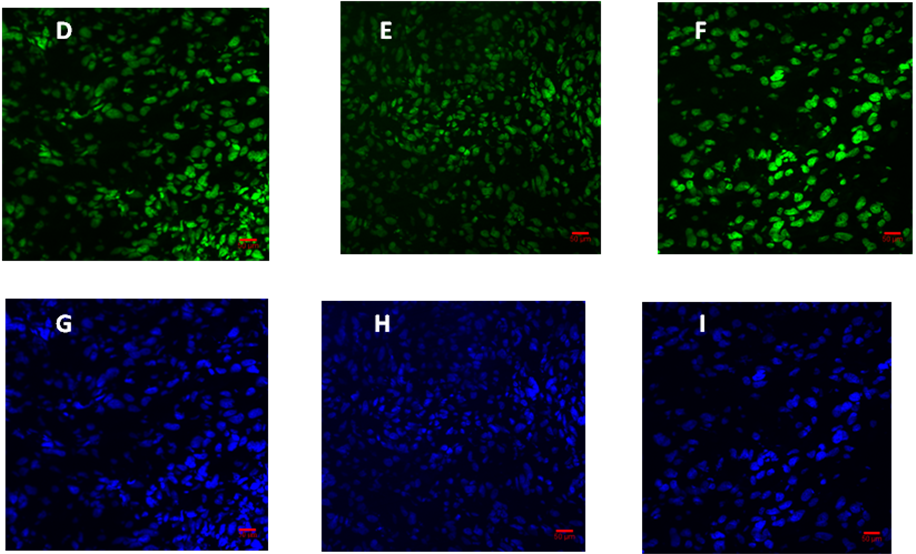


**S1 Fig B. ELISA standard curve for 8-HO-dG level investigation.**

**
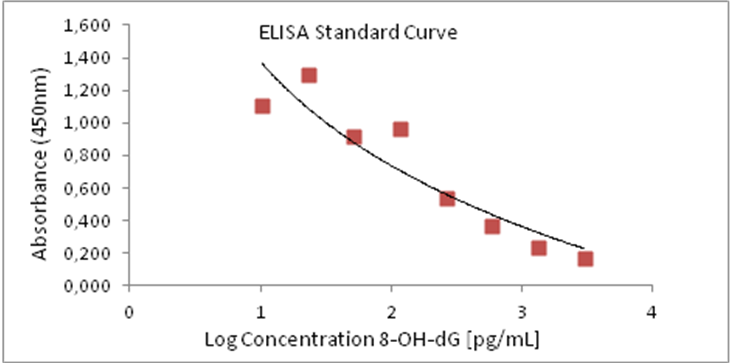
**

**S1 Table F. Formation of 8-HO-dG in nuclear DNA of human gliblastoma multiforme tumour tissue cells treated with platinum nanoparticles and cisplatin.**

| **Group** | **Average of Absorbance** | **Average of Absorbance - blank** | **Calculation 8-OH-dG by standard curve equation** | **Log(concentration) pg/mL** | **Final concentration [Multiply by delution][pg/mL]** |
| --- | --- | --- | --- | --- | --- |
| Blank | 0.024 | 0.024 |  |  |  |
| Control | 1.238 | 1.214 | 1.154 | 14.3 | 142.5 |
| Control | 1.168 | 1.144 | 1.302 | 20.0 | 200.5 |
| Control | 1.169 | 1.145 | 1.300 | 20.0 | 199.6 |
| NP-Pt | 1.022 | 0.999 | 1.610 | 40.7 | 407.4 |
| NP-Pt | 1,032 | 1,008 | 1,591 | 39,0 | 389.7 |
| NP-Pt | 1.052 | 1.028 | 1.547 | 35.3 | 352.6 |
| CisPt | 1.156 | 1.132 | 1.328 | 21.3 | 212.9 |
| CisPt | 1.174 | 1.150 | 1.290 | 19.5 | 195.0 |
| CisPt | 1.164 | 1.140 | 1.311 | 20.4 | 204.4 |
| B0 | 1.294 | 1.270 | - | - | - |
| TA | 1.839 | 1.816 | - | - | - |
| NSB | 0.003 | - | - | - | - |
| **B0-NSB** | **1.291** | **-** | **-** | **-** | **-** |

Abbreviations: NP-Pt – platinum nanoparticles, CisPt – cisplatin.
